# Pasteur and His Discoveries

**Published:** 1896-01

**Authors:** 


### Abstracts and Selections.

### Pasteur and His Discoveries.

In the chorus of eulogy that has poured forth in honor of M. Pasteur since his death, there has been but one discordant note—that of Henri Rochefort, who has never had any faith in the scientist's preventive hydrophobia. On the day after Pasteur's death he printed the following editorial in *L' Intransigéant*, under the title "To Each His Work":

"It would be in a high degree unjust to deny to Pasteur his scientific sincerity, his probity, and his disinterestedness. None the less true is it that the two chief titles on the strength of which humanity's gratitude is claimed for him—the microbian theory and the discovery of the anti-rabic virus—belongs to him only in a very limited degree.

"The idea of seeking in the development or propagation of special microbes the cause of contagious maladies, epidemic or simply endemic, was first conceived, not by Pasteur, but by his forerunner, Raspail, who proposed to destroy the invisible and impalpable vermin, then known as animalcules, by the use of camphor and other antiseptics.

"Raspail, who was a socialistic Republican, and had conspired with the principal revolutionists of his time, was treated as a fanatic and a dreamer by the entire medical body, whose members would have regarded their future as compromised, or their practice as ruined if they had pretended to take seriously the principles laid down by a man who had refused the cross of honor offered him by Louis Philippe.

"The day when Raspail undertook, and with success, to give fever to the occupants of a chamber by placing in the window a basin of water and leaving it there to stagnate, the microbian theory was found, and the pharmacies which he established in Paris, had no other object than the sale of remedies destined to kill bacilli.

"If there had been no Pasteur, the German doctor, Behring, whose discovery the French doctor, Roux, has taken up and perfected, would probably have invented all the same the virus against croup. If there had been no Raspail, perhaps the question would still be in suspense.

"Certainly Pasteur has extended the domain of the investigations begun by Raspail. He has generalized them to the point of applying them to the cure of sheep and other animals; he has 'Pasteurized' drinks. But it was Raspail's genius that gave birth to the idea of such medication.

"As to the manufacture of anti-rabic bouillon based on Jenner's homeopathic system of preventing small-pox—*similia similibus*—no one having a name in the scientific world would dare to affirm that it has given definitive results. I do not know whether Pasteur's complaint, made to several of his friends, that the more ambitious among those about him went too fast with his discovery and inoculated people in spite of him, has any foundation; but there is seldom a period of three months in which some man who has been bitten by a mad dog does not die in the convulsions of hydrophobia, a few weeks after having been treated at the Pasteur Institute.

"Only recently an unfortunate was seized, on leaving there, with a fit of madness so violent that he tried to bite every one who approached him. The learned Dr. Peter, a member of the Academy of Medicine, has kept an account of the failures of the Pasteur method, and has easily succeeded in demonstrating that the number of deaths from hydrophobia has increased rather than diminished since the substitution of inoculation for immediate cauterization.

"Ten or twelve Russian peasants who had been bitten by a mad wolf came to Paris to be treated at the famous Institute. Shortly after their return to their native land, all of them died of hydrophobia.

"A father and mother whose little daughter had been slightly wounded by the tooth of a dog suspected of madness, took her to Pasteur by the advice of their neighbors, although the child showed none of the symptoms of the frightful malady. A month and a half after having been placed under the influence of the virus, she fell sick and died, foaming at the mouth.

"These grief-stricken parents came themselves to tell me of their frightful misfortune, which they had no hesitation in attributing to the remedies that had been applied to their daughter. Yet, as is usually the case, it is not his great and splendid work in rendering meat, drinks, and provisions wholesome, but his pretended discovery of the anti-rabic virus, that has given Pasteur his popularity. To-morrow he will have a national funeral, and among those who will march in the procession very few will know aught of the dead save his bouillon of rabbit's brains."—*The Literary Digest.*

[The "Red-Back" long ago recorded its entire unbelief in the whole theory and practice.]

